# Identifying critical nodes in temporal networks by network embedding

**DOI:** 10.1038/s41598-020-69379-z

**Published:** 2020-07-27

**Authors:** En-Yu Yu, Yan Fu, Xiao Chen, Mei Xie, Duan-Bing Chen

**Affiliations:** 1 Big Data Research Center, University of Electronic Science and Technology of China, Chengdu, 611731 People’s Republic of China; 2 Information Assurance Office of Army Staff, Beijing, 100043 People’s Republic of China; 3 The Center for Digitized Culture and Media, University of Electronic Science and Technology of China, Chengdu, 611731 People’s Republic of China; 4 Union Big Data Tech. Inc., Chengdu, 610041 People’s Republic of China

**Keywords:** Information theory and computation, Computer science

## Abstract

Critical nodes in temporal networks play more significant role than other nodes on the structure and function of networks. The research on identifying critical nodes in temporal networks has attracted much attention since the real-world systems can be illustrated more accurately by temporal networks than static networks. Considering the topological information of networks, the algorithm *MLI* based on network embedding and machine learning are proposed in this paper. we convert the critical node identification problem in temporal networks into regression problem by the algorithm. The effectiveness of proposed methods is evaluated by SIR model and compared with well-known existing metrics such as temporal versions of betweenness, closeness, k-shell, degree deviation and dynamics-sensitive centralities in one synthetic and five real temporal networks. Experimental results show that the proposed method outperform these well-known methods in identifying critical nodes under spreading dynamic.

## Introduction

Complex networks are common in real life and can be used to represent complex systems in many fields^1^. Identifying critical nodes is an important topic in complex networks and it plays a crucial role in many applications, such as market advertising, rumor controlling and valuable scientific publication predicting^2,3^. In recent years, many methods are proposed to measure the importance of nodes in static networks. Among of these, degree centrality^4^, semi-local centrality^5^, k-shell^6^ and H-index^7,8^ are based on nodes’ degrees; closeness^9^, betweenness^10^ and eccentricity centralities^11^ are based on paths in networks; and PageRank^12^, LeaderRank^13^ and HITs^14^ are based on eigenvector. However, the static networks whose edges are always active can not illustrate the dynamical systems. For this case, temporal networks are more suitable to present them^15–17^. Compared with static networks, temporal networks contain time information. The topology of temporal networks are changing with time and can describe many important activities in the real-word, including face-to-face conversations^18^, the outbreak of epidemics^19^ and the spread of news and ideas. Same as static networks, identifying critical nodes or influential spreaders on temporal networks also is a hot research topic. Up to now, there have been many methods focus on this problem. Kim et al. have presented a simple yet effective model, the time-ordered graph, which reduces a dynamic network to a static one with directed flows and defined the temporal version of degree, closeness and betweenness on temporal networks^20^. Taylor et al. proposed the eigenvector-based centrality for temporal networks^21^, where the eigenvector and its components of a supra-centrality matrix can reflect the importance of nodes. Liu et al.^22^ proposed dynamic-sensitive centrality to measure the importance of nodes in static networks and temporal dynamic-sensitive centrality^23^ has advantage over the method proposed by Liu et al, which based on Markov chain for the epidemic model and derive the analytical result of node influence.

Although there are many methods to measure the influence of nodes in static or temporal networks, most of them can be regarded as a mission to find what kind of structure will make the node more influential. In this paper, we convert the critical node identification problem in temporal networks into regression problem by network embedding^24–26^. Network embedding assigns nodes in a network to low-dimensional representations and effectively preserves the network structure^27^. Recently, significant progresses have been made toward this emerging network analysis paradigm^28^. And network embedding has been used in community detection^29,30^, link prediction^17^ and etiology of diseases^31^. In terms of node embedding, Niepert et al. proposed a framework for learning convolutional neural networks for arbitrary graphs^32^, presenting a general approach to extract locally connected regions from networks. *node*2*vec*^33^ is an algorithmic framework for learning a low-dimensional representations of nodes in complex networks and the mapping of nodes to a low-dimensional space of features can be learned by maximizes the likelihood of preserving network neighborhoods of nodes. Kipf et al. presented a scalable approach for semi-supervised learning on complex networks which is based on an efficient variant of convolutional neural networks^34^. In order to explore the impact of temporal information on the importance of the nodes, Qu et al.^35^ proposed a temporal information gathering (TIG) process for evaluating the significance of the nodes in temporal networks. The key to the TIG process is that the importance of a node depends on the importance of its neighborhood. Qi et al.^36^ presented a Deep Autoencoding Gaussian Mixture Model (DAGMM) for unsupervised anomaly detection. In DAGMM, low-dimensional representations of nodes are generated by a deep autoencoder and applied to rank nodes in temporal networks.

By using the topological information, we proposed the algorithm based on network embedding and machine learning. The performance of proposed method is compared with that of temporal versions of betweenness centrality, closeness centrality^37^, k-shell^38^, degree deviation centrality^39^ and dynamics-sensitive centrality^23^ in SIR model^40,41^ for one synthetic and five real temporal networks. The results show that the proposed method in this paper can effectively identify critical nodes which have greater impacts on information spreading in temporal networks.

## Results

The real spreading ability of a node is estimated by SIR spreading model in this paper. The performance of *MLI* is evaluated by SIR spreading model, and compared with well-known existing metrics such as temporal versions of betweenness centrality, closeness centrality, k-shell, degree deviation centrality and dynamics-sensitive centrality in one synthetic and five real temporal networks. In all experiments, we set $D=8, \alpha =0.2$.

### Data sets

**Training Data Set.** A temporal scale-free network generated by BA model^42^. In addition, using other network generation models can also get good training results. We use the BA model in this paper, and other models can be used, such as WS models^43^, etc.

**Testing Data Sets.** Six temporal networks are used to evaluate the performance of the methods.

1. Temporal scale-free network(TSF). This undirected network is a combination of 30 snapshots, and each snapshot is generated by BA model^42^.
2. High school friendship relations network(FRI). This undirected network is a medium-sized data set correspond to the contacts and friendship relations between students in a high school^44^.
3. Contact network(Contact). An undirected network represents contacts between users measured by carried wireless devices^45^.
4. Sociopatterns-hypertext network(Hypertext). This is an undirected network of face-to-face contacts of the attendees of the ACM Hypertext 2009 conference^46^.
5. DNC co-recipient network(DNC). This is the undirected network of emails in the 2016 Democratic National Committee email leak^47^.
6. UC Irvine messages network(UCS). An undirected network contains sent messages between the users of an online community of students from the University of California, Irvine^48^.

In Table 1, some detailed statistical properties of above networks are listed. In this paper, these networks are both undirected and unweighted. FRI, Contact, Hypertext, DNC, UCS are real-world networks which represent human interactions in diverse social systems and have different topological and temporal characteristics, Training and TSF are generated by BA model. All temporal networks are divided into 30 snapshots.

**Table 1:** Statistical properties of the networks used in the experimental analysis. *N* and *E* are the total number of nodes and edges, respectively. *L* is the number of time points and $\delta$ is the time interval.

Network	*N*	*E*	*L*	$\delta$
Training	1000	29940	30	*N*/*A*
TSF	500	14970	30	*N*/*A*
FRI	180	45070	30	2 hours
Contact	274	28244	30	10 minutes
Hypertext	410	20818	30	12 hours
DNC	2029	39264	30	16 days
UCS	1899	59835	30	6 days

### Spreading performance

**Figure 1:**
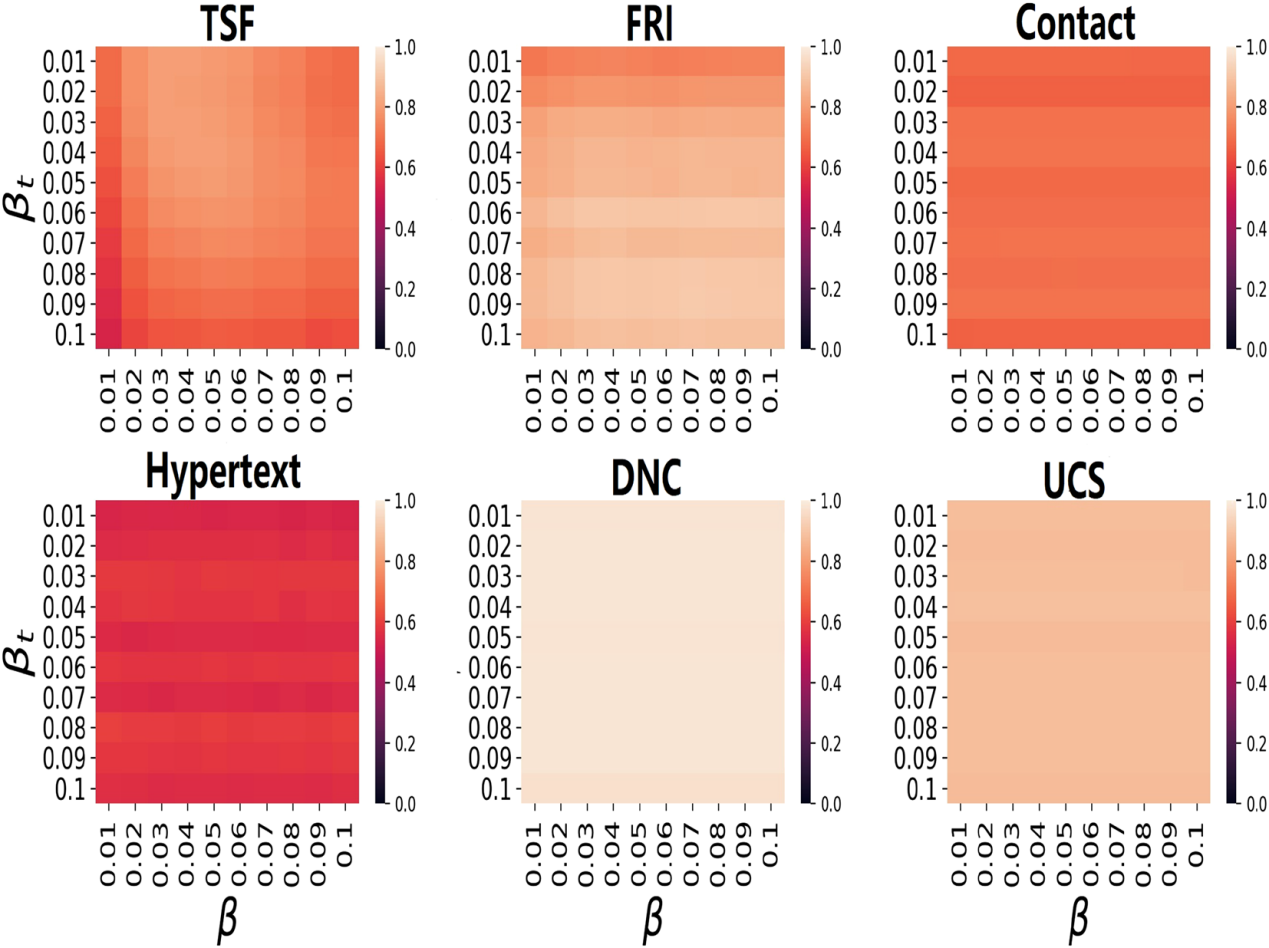
The heat map of kendall’s tau correlation coefficients between the ranking scores and real infected scales with varying infection rate $\beta$ and train infection rate $\beta _{t}$ by *MLI*.

In order to evaluate the performance of *MLI* on different $\beta$ and $\beta _{t}$. The kendall’s tau coefficients between the ranking scores and the real infected scales on different $\beta$ and $\beta _{t}$ is shown in fig. 1. From fig. 1, it can be seen that there is a high value in any position of these heat maps. When infection rate $\beta$ and train infection rate $\beta _{t}$ varies from 0.01 to 0.10, the performance of *MLI* will vary slightly with the change of $\beta _{t}$.

**Figure 2:**
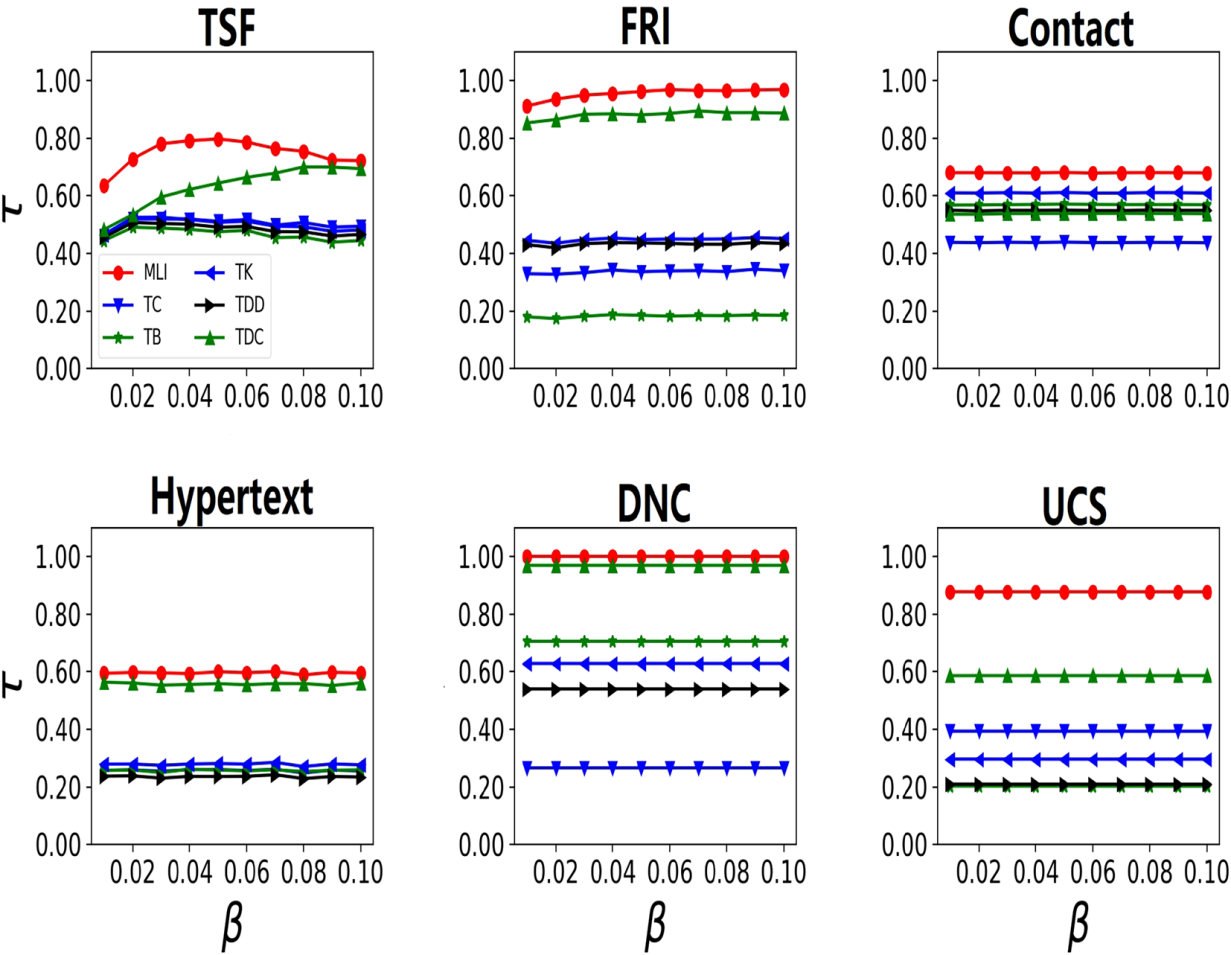
kendall’s tau correlation coefficients between the ranking scores and the relative differences of real infected scales with varying infection rate $\beta$.

As mentioned in above analysis, *MLI* performs well in most cases with slight influence of train infection rate $\beta _{t}$. So we can fix train infection rate $\beta _{t}$ as 0.1 in the following analysis. In order to evaluate the performance of these algorithms under different infection rates, *MLI* is compared with other five methods. From fig. 2, it can be seen that *MLI* has the maximum value under the infection rate varying from 0.01 to 0.1. It means that the ranking result of *MLI* is the closest to that of the real infected scales in all methods.

**Figure 3:**
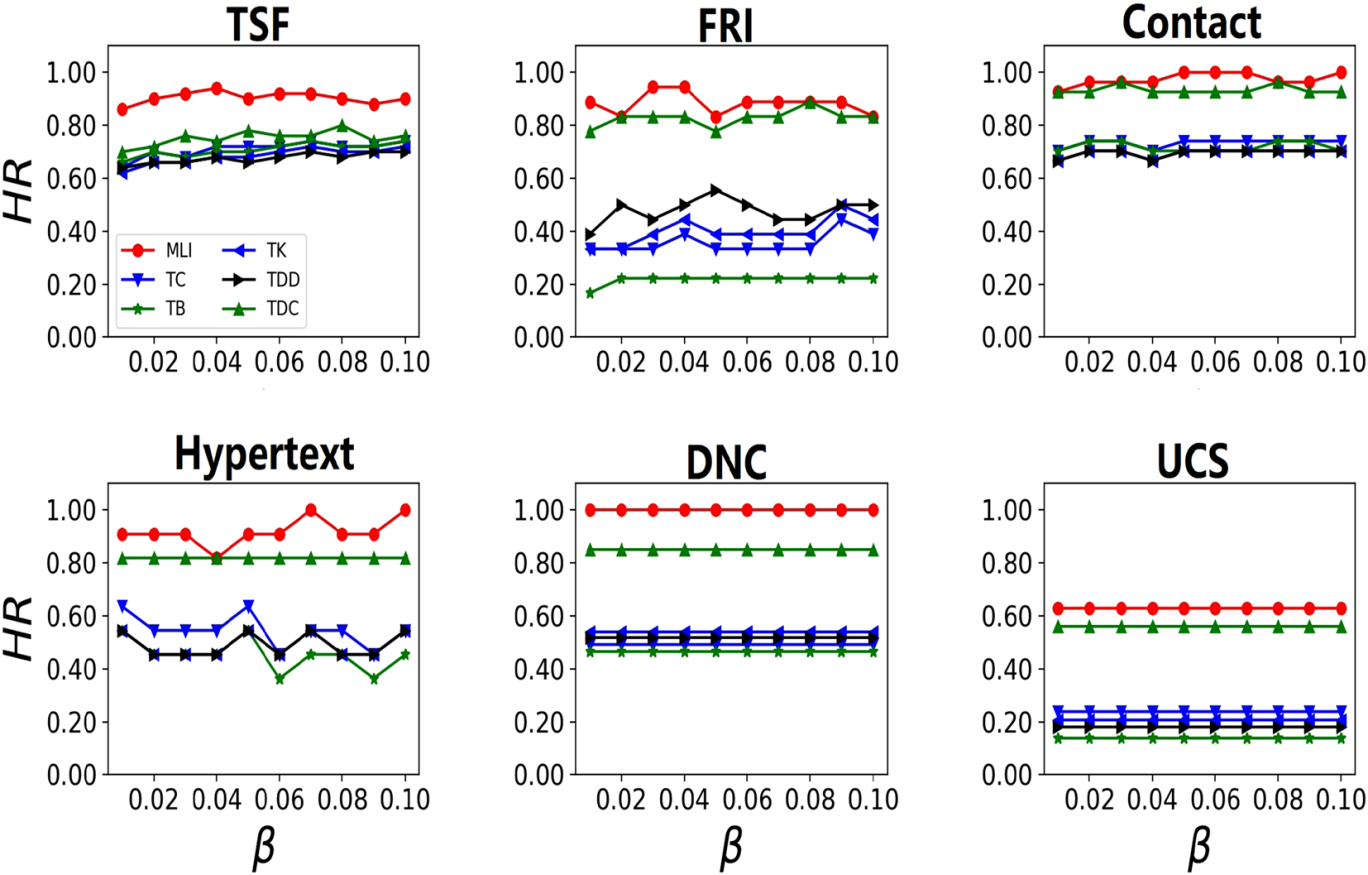
The top10% *HR* under different infection rate $\beta$.

We also use the top *k* comparison method in this paper since we often care about top-ranked nodes. For details, sort nodes in descending order according to their ranking score firstly, and compare the top *k* nodes obtained by methods with real top *k* nodes simulated by SIR spreading model. The evaluation index is hitting rates(*HR*), which is defined as

1 $$\begin{aligned} HR = \frac{|C\bigcap R|}{|R|}, \end{aligned}$$

where *C* and *R* are top *k* nodes obtained by algorithms and SIR spreading model respectively and $\left| \cdot \right|$ is the size of set. The higher the *HR* is, the better the performance of the algorithm is. From fig. 3, it can be seen that *MLI* has the maximum *HR* in most cases when find the top 10% critical nodes under the infection rate varying from 0.01 to 0.1. These results demonstrate that *MLI* can find critical nodes under different infection rates.

## Discussion

How to identifying critical nodes in temporal networks is an interesting and important topic in many applications. Most of previous researches concentrate on finding what kind of structure will make the node more influential. Inspired by the concept of network embedding and machine learning, the algorithm *MLI* is proposed in this paper. According to the experimental results on five real-world and a synthetic temporal networks, *MLI* performs much better than other five benchmark methods in identifying the nodes which have great impact on information spreading. We can use *MLI* to detect the potential super-spreaders for epidemic control in temporal networks. *MLI* enable us to investigate the dynamics in spreading process and the results of parameters analysis show that *MLI* outperform other five methods significantly in most cases with fixing train infection rate $\beta _{t}$. What’s more, *MLI* has a low computational complexity and can be used in large-scale networks. The method presented in this paper have provided a new idea for identifying critical nodes in complex networks, and the method can be extended to many other dynamical analyses such as the impact of edges on spreading dynamics.

## Methods

### Temporal networks

**Figure 4:**
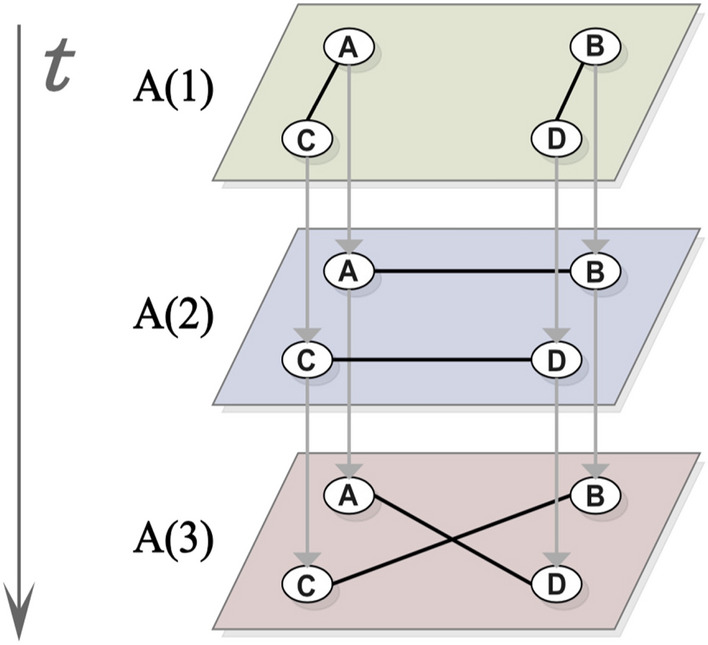
An example temporal network represented by using a multilayer network structure.

A temporal network with *N* nodes and time length *T* can be defined as a sequence of *L* snapshots with $\delta = T/L$. That is, a temporal network can be represented as a series of static graphs $G_{1},G_{1},\ldots ,G_{L}$. Where $G_{t}\left( 1\leqslant t\leqslant L \right)$ represents the aggregate graph which consists of *N* nodes and a set of edges $E_{t}$ where an edge $e(u, v) \in E_{t}$ only if node *u* has a connection with node *v* at snapshot *t* (a time interval $[\delta (t-1), \delta t]$). In this study, all edges are undirected. Fig. 4 is an example temporal network represented by a multilayer network structure. There are four nodes A, B, C, D with *T* = 3, *L* = 3 and $\delta = 1$.

### Convolutional Neural Networks

CNNs are kind of Feedforward neural networks with convolutional computation and deep structure. It is one of the representative algorithms of deep learning. CNN is an incredibly successful technology that has been applied to computer vision and natural language processing^49,50^. CNNs in essence are neural networks which use the convolution operation as one of their layers. A typical CNN consists of several convolution and pooling layers. The purpose of the first convolutional layer is to extract a common pattern found within localized regions of the input data. CNNs convolve learned filters over the input data, computing the inner product and outputting the result as tensors whose depth is the number of filters.

### Benchmark methods

We consider some benchmark methods in this paper, Tang et al.^37^ proposed a method to identify important nodes using temporal versions of conventional centrality metrics, Kim et al.^20^ extend Tang’s work to a more general and more realistic model. Readers can refer to Tang et al.^37^ or Kim et al.^20^ for details of the temporal versions of conventional centrality. In this paper, we consider *TC* and *TB* proposed by Kim et al.^20^, *TK* proposed by Wang^39^, *TDD* proposed by Ye^38^ and *TDC* proposed by Huang^23^. For all methods, we calculate the value in a time interval [0, *T*] with *L* time snapshots.

The temporal version of closeness centrality(*TC*) is defined as

2 $$\begin{aligned} TC(v) = \sum _{0\leqslant t< T}^{}\sum _{u\in V\setminus \{v\}}^\frac{1}{\Delta _{t, T}(v, u)} \end{aligned},$$

where $\Delta _{t, T}(v, u)$ is the distance of temporal shortest path from node *v* to node *u* on a time interval [*t*, *T*]. If there is no temporal path from *v* to *u* on a time interval [*t*, *T*], $\Delta _{t, T}(v, u)$ is infinity.

The temporal version of betweenness centrality(*TB*) is defined as

3 $$\begin{aligned} TB(v) = \sum _{0\leqslant t< T} \sum _{\begin{array}{c} s\ne v\ne d \in V \\ \sigma _{t, T}(s, d) > 0 \end{array}} \frac{\sigma _{t, T}(s, d, v) }{\sigma _{t, T}(s, d)} \end{aligned},$$

where $\sigma _{t, T}(s, d)$ denotes the number of temporal shortest paths from source *s* to destination *d* on the time interval [*t*, *T*], $\sigma _{t, T}(s, d, v)$ is the number of paths from source *s* to destination *d* on the time interval [*t*, *T*] with *v* in their interior.

The temporal k-shell(*TK*) is defined as

4 $$\begin{aligned} TK(v)= \sum _{u\in \Gamma _{v}}\sum _{t=1}^{L}min\left\{ k _{s}^{t}(v), k_{s}^{t}(u)\right\} \end{aligned},$$

where $k _{s}^{t}(v)$ denote the k-shell score of node *v* in the time snapshot *t*, $\Gamma _{v}$ is node *v*’s neighbors in the slice network.

The temporal degree deviation centrality(*TDD*) is defined as

5 $$\begin{aligned} TDD(v)=\sqrt{\frac{1}{L}\sum _{t=1}^{L}(D_{t}(v)- D(v))^{2}} \end{aligned},$$

where $D_{t}(v)$ denote the degree of node *v* in the time snapshot *t*, *D*(*v*) is the average number of $D_{t}(v)$ in all slice networks.

The temporal dynamics-sensitive centrality(*TDC*) of all nodes can be described by the vector

6 $$\begin{aligned} S= & {} \sum _{r=0}^{L-1}\beta H_{*}^{r}A(r+1)V \end{aligned},$$

7 $$\begin{aligned} H_{*}^{t}= & {} \prod _{\alpha =t}^{1}[\beta A(\alpha )+(1-\mu )I], H_{*}^{0} = 1 \end{aligned},$$

where *S* is a vector and denote the spreading influence of all nodes, *A*(*t*) is the adjacency matrix in time snapshot *t*, $V=(1,1,\ldots ,1)^{T}$, $\beta$ and $\mu$ are infection probability and recover probability in SIR spreading model.

### SIR spreading model

In SIR model^51^, there are three states: (1) *S*(*t*) denotes the number of nodes which may be infected(not yet infected); (2)*I*(*t*) denotes the number of nodes which have been infected and will spread the disease or information to susceptible nodes; (3)*R*(*t*) denotes the number of nodes which have been recovered from the disease or boredom the information and will never be infected by infected nodes again. In a network, each infected node will infect all susceptible neighbors with a certain probability $\beta$. Infected nodes will be recovered with probability $\mu$(for simplicity, $\mu =1$ in this paper)at each step. The process is repeated within the given time step *t*($t\leqslant$L). $N_{v}(t)$ is defined as the number of infected nodes after *t* steps under the disease spreads from the initial node *v* firstly. We can use $N_{v}(L)$ to represent the finally infected scale of node *v* in this paper.

### MLI model

With the development of machine learning, some researchers have proposed the concept of network embedding, which aims at learning low-dimensional latent representation of nodes in a network. These representations can be used as features for a wide range of tasks on graphs such as classification, clustering, link prediction and visualization. Inspired by this concept, we combine network embedding and machine learning to identify critical nodes in temporal networks. For details, the method *MLI*(machine learning index) can be described by following steps:

1. Feature Matrices and Labels: Similar to convolutional neural network for images, we need to input feature matrices and labels to training model. For each node, we construct a feature matrix by its neighborhoods in all snapshots. And obtaining all nodes’ infected scale by SIR spreading model(training infection rate is $\beta _{t}$ and training recovery rate is $\mu _{t}= 1$) as the labels. For details, the node embedding algorithm is shown in Algorithm. 1. When finding neighbors, nearer neighbors have higher priority to choose, that is, 1-hop neighbors have priority over 2-hop neighbors, if two nodes have equal hops, the node with larger degree has been chosen preferential.
2. Convolutional Architecture: The convolutional neural network in this paper have 2 convolutional layers, 2 pooling layers and 1 fully-connected layer. In the first convolutional layer, kernel size is $5 \times 5$, input channel is 1 and output channels are 16, stride is 1 and paddings are 2. In the second convolutional layer, kernel size is $5 \times 5$, input channels are 16 and output channels are 32, stride is 1 and paddings are 2. 2 pooling layers are $2 \times 2$ max pooling and the fully-connected layer is $32*(D/4)*(D/4) \times 1$. The activation function is ReLU and the loss function is squared loss function.
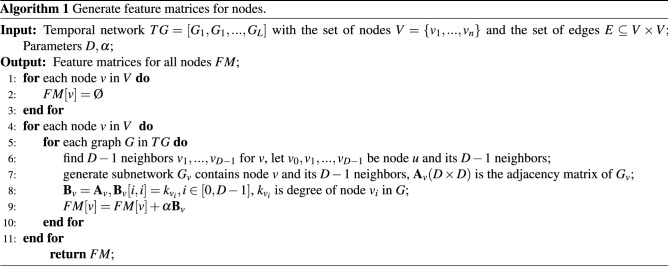
3. Ranking Nodes: Firstly, use training sets (generate feature matrices and labels for all nodes) to train the CNN model. Secondly, select arbitrary networks, generate feature matrices for all nodes without labels and get the score of these nodes by the trained CNN model. Finally, ranking the nodes by its score.

### Time complexity

We compare the time complexity of the above six methods. Although *MLI* needs time to generate training set and train the parameters, we can use the parameters for all temporal networks. Let the size of feature matrices be $D \times D(D<<N)$, the temporal network has *N* nodes and *L* snapshots. The time complexity of generating feature matrices is *O*(*NL*),the time complexity of training^52^ is $O(I\cdot \sum _{l=1 }^{P}M^{2}_{l}\cdot K^{2}_{l}\cdot C_{l-1}\cdot C_{l})$, where *I* is the number of iterations. *P* is the number of convolutional layers. $M_l$ is the side length of the output feature maps of convolutional kernels at the *l*^th^ convolutional layer. $K_l$ and $C_l$ are the side length of convolutional kernels and the number of output channels at the *l*^th^ convolutional layer, respectively. So the time complexity of *MLI* in new temporal networks is $O(NL+\sum _{l=1 }^{P}M^{2}_{l}\cdot K^{2}_{l}\cdot C_{l-1}\cdot C_{l})$.

In addition, the time complexity of *TDD* is *O*(*m*). We need take *O*(*m*) to calculate the k-shell score of all nodes in all snapshots. So the time complexity of *TK* is also *O*(*m*). And the time complexity of *TDC* is $O(n^{2})$ because we need take $O(n^{2})$ to calculate the multiplication of sparse matrices. The time complexity of *TC* is $O(mn^{2})$ and the time complexity of *TB* is $O(m^{3}n^{3})$^20^. From above analysis, it can be seen that $MLI_{LM}$ has a low computational complexity and can be used in large-scale networks.
